# Time is vision: a systematic review of urgent venous sinus stenting for fulminant idiopathic intracranial hypertension

**DOI:** 10.3389/fneur.2026.1824887

**Published:** 2026-04-30

**Authors:** Amol Mehta, Daryl Goldman, Brandon Philbrick, Jonathan Sisti, Mark J. Kupersmith, Christopher P. Kellner, Michael Travis Caton

**Affiliations:** 1 Department of Neurosurgery, Icahn School of Medicine at Mount Sinai, New York, NY, United States; 2 Department of Ophthalmology, New York Eye and Ear Infirmary, Union Square Eye Care, Icahn School of Medicine at Mount Sinai, New York, NY, United States

**Keywords:** idiopathic intracranial hypertension, IIH, stenting, vision, venous sinus stenosis, fulminant, papilledema, endovascular

## Abstract

We systematically evaluated the evidence for urgent venous sinus stenting (VSS) as a sight-saving strategy in fulminant idiopathic intracranial hypertension (IIH), a rapidly progressive phenotype with imminent risk of irreversible visual loss. A comprehensive search of PubMed, Embase, Web of Science, and Scopus identified reports of patients meeting fulminant IIH criteria who underwent VSS as an urgent intervention; eligible studies required detailed clinical characterization and post-stenting visual or papilledema outcomes. Seven studies published between 2015 and 2023, comprising 23 patients, met inclusion criteria. Across cohorts, patients were young, predominantly female, and uniformly presented with severe papilledema and rapidly declining visual function, frequently after failure or intolerance of maximal medical therapy and/or cerebrospinal fluid diversion. All patients had dural venous sinus stenosis with a documented trans-stenotic pressure gradient prior to treatment and underwent technically successful stent placement. Urgent VSS was associated with high rates of papilledema resolution or marked improvement. There was stabilization or improvement of visual acuity and visual fields in nearly all reported cases, and substantial relief of headache and pulsatile tinnitus where documented. Need for subsequent shunting or optic nerve sheath fenestration was uncommon, and reported complications were infrequent and non-catastrophic, with no procedure-related mortality. Although limited by small sample size, retrospective design, and heterogeneity in fulminant definitions and outcome reporting, the available data support urgent VSS as a rational, venous outflow–targeted, and potentially vision-preserving option in carefully selected fulminant IIH with venous sinus stenosis, warranting prospective, comparative evaluation.

## Introduction

Idiopathic intracranial hypertension (IIH) is diagnosed by the modified Dandy criteria where a patient demonstrates signs and symptoms of elevated intracranial pressure (ICP) without an underlying mass lesion or venous sinus thrombosis or secondary to exogenous drugs, which typically presents with headaches and pulsatile tinnitus (1). In severe cases, IIH causes papilledema and carries risk of permanent vision loss (1). “Fulminant IIH” refers to an acute, rapidly progressive form of IIH with severe visual deterioration developing over days to a few weeks (2). This rare subtype (occurring in ~2–3% of IIH cases) is a neuro-ophthalmologic emergency due to the high risk of irreversible blindness if not promptly treated. The formal definition often used is onset of severe visual loss within 4 weeks of symptom onset (2) though some authors extend the timeframe up to 8 weeks for “acute IIH” if there is demonstrably rapid decline (3). In practice, any IIH patient with rapidly worsening vision over days to a few weeks is considered fulminant and warrants urgent intervention (4).

Standard management of fulminant IIH comprises intensive medical therapy (acetazolamide) and surgical procedures such as permanent or temporary CSF diversion (lumbar drain, external ventricular drain, lumboperitoneal or ventriculoperitoneal shunts) or optic nerve sheath fenestration (ONSF) (1). Although not prospectively tested in a fulminant IIH cases series, the benefit of acetazolamide seems to take about a month to take effect and weight loss is even a slower process. Traditional management algorithms recommend hospitalization for high-dose medical therapy and serial neuro-ophthalmologic assessment, often with temporizing CSF diversion such as serial lumbar punctures or external CSF drains, or even intravenous steroids are employed as a bridge to definitive surgery (4, 5) (shunt or ONSF) to lower ICP and protect vision rapidly (2, 4). These options have major limitations: while CSF shunts can effectively lower ICP and alleviate both headache and papilledema, they require open surgery and are likely to require revision procedures over the lifetime of the patient (6). ONSF can directly relieve optic nerve head pressure and often stabilize or improve vision, but typically does not relieve headaches or generalized symptoms (7, 8). Moreover, ONSF carries its own risks (up to ~40% complication rate, including fenestration closure and rare visual loss from central retinal artery occlusion) (8).

Over the past two decades, venous sinus stenting (VSS) has emerged as an alternative, minimally invasive treatment for IIH patients with venous sinus stenosis and a trans-stenotic pressure gradient (9). Transverse sinus stenosis is found in up to 90% of IIH patients and is thought to create a secondary venous outflow obstruction that further raises intracranial venous pressure and impedes CSF resorption (10, 11). Venous sinus stenting augments cranial venous drainage, eliminating or reducing the trans-stenotic pressure gradient with a corresponding reduction in ICP (12, 13). Multiple case series and meta-analyses in predominantly chronic, medically-refractory IIH have reported high success rates of VSS in reducing ICP and improving headaches, tinnitus, papilledema, and visual function with prior literature suggesting rates of papilledema improvement of 89%, visual symptoms in 88%, headaches 49% and tinnitus in 95% (9, 14, 15). The overall serious complication rate of VSS in elective cases is low, and the vast majority of IIH patients do not require further shunt surgery after stenting (16, 17). These advantages make VSS an attractive option, especially for fulminant cases where rapid, complete relief of venous outflow obstruction could rescue vision without the morbidity of open surgery.

Notably, VSS may be particularly valuable in fulminant IIH to rapidly preserve vision in the acute period when time is of the essence (18, 19). However, evidence specifically for emergent VSS in fulminant IIH has been limited to small series and reports. The relatively slow adaptation of VSS to cases of fulminant IIH may be due to the necessity of dual antiplatelet therapy (DAPT) to maintain stent patency, which subsequently would make surgical CSF diversion procedures risky to perform shortly after initiating DAPT. The objective of this study is to critically appraise the efficacy, outcomes, safety of VSS in fulminant IIH through systematic review of the literature.

## Methods

A comprehensive literature search was conducted across PubMed, Embase (Ovid), Scopus, and the Cochrane Central Register of Controlled Trials (CENTRAL) from January 2015 to October 2025. This systematic review was conducted and reported in accordance with the Preferred Reporting Items for Systematic Reviews and Meta-Analyses (PRISMA) 2020 guidelines. A completed PRISMA checklist is provided as Supplementary Material. The review protocol was not prospectively registered. The search combined terms related to fulminant idiopathic intracranial hypertension, malignant idiopathic intracranial hypertension, acute vision loss, and venous sinus stenting. In PubMed, the following combination was used: (“fulminant” OR “malignant” OR “acute”) AND (“idiopathic intracranial hypertension” OR “pseudotumor cerebri”) AND (“venous sinus stenting” OR “stent”). Similar terms and equivalent syntax were applied to Ovid Embase, Scopus, and Cochrane CENTRAL. Reference lists of key studies and reviews were also manually screened to identify additional eligible articles.

### Inclusion and exclusion criteria

Studies were considered eligible if they described patients with fulminant idiopathic intracranial hypertension (IIH), defined as objective rapid visual decline or vision loss occurring within 4 weeks of symptom onset. We acknowledge that no single universally accepted operational definition of fulminant IIH exists in the literature. For the purposes of this review, we adopted an inclusive working definition encompassing rapid visual decline occurring within approximately 4 to 8 weeks of symptom onset, requiring urgent intervention, consistent with the range of definitions used across the included studies. Only studies that reported outcomes following venous sinus stenting (VSS) performed as an urgent or emergent intervention were included. Eligible publications were limited to English-language, peer-reviewed articles that provided extractable clinical data, such as visual testing and papilledema measurements pre- and post-intervention, or venous pressure gradients.

Studies were excluded if they were narrative or systematic reviews, editorials, or non–peer-reviewed abstracts, if they were published in languages other than English, or if they focused on non-fulminant IIH, alternative etiologies such as intracranial hypotension or mass lesions, or procedures other than VSS (e.g., optic nerve sheath fenestration or shunting). Case reports lacking detailed outcome data specific to fulminant IIH and animal or laboratory investigations were also excluded.

### Study selection

All retrieved records were imported into a single reference library, and duplicate entries were removed using both automated and manual verification. Two reviewers independently screened titles and abstracts for relevance. Full texts of potentially eligible studies were then assessed for inclusion using the pre-specified criteria. Disagreements were resolved by consensus.

Out of 88 total records identified across all databases (PubMed = 25, Embase = 40, Scopus = 22, Cochrane = 1), 43 unique articles remained after deduplication. After title and abstract screening, 36 records were excluded for reasons including non-original data, conference abstracts, non-English language, or unrelated topics. 7 studies met full eligibility criteria and were included in the final qualitative synthesis (Supplementary Figure S1).

### Data extraction and synthesis

From each included study, the following data were extracted: study design, sample size, patient demographics, definition of fulminant IIH, stenting indication and technique, visual and papilledema outcomes, and follow-up duration. Given the heterogeneity of study designs (case series and case reports), results were synthesized qualitatively. The primary outcomes of interest were visual recovery, papilledema resolution, and stabilization of visual function following VSS as compared to pre-intervention testing. Secondary data such as venous pressure gradients and complications were also noted. We note that clinical and visual outcomes were heterogeneously defined across studies, with variable use of quantitative vs. qualitative measures and inconsistent follow-up durations, which precluded formal meta-analysis and limited the robustness of cross-study comparisons.

### Quality assessment

Study quality was assessed using the Joanna Briggs Institute (JBI) critical appraisal checklists for case reports and case series, which evaluate methodological rigor across domains including clarity of patient selection criteria, completeness of clinical condition reporting, validity of outcome assessment methods, adequacy of follow-up, and appropriateness of statistical analysis. Each included study was independently appraised by two reviewers, and discrepancies were resolved by consensus. Given the absence of controlled studies, formal risk-of-bias tools designed for randomized or comparative designs were not applicable. A summary of the quality appraisal is provided in Supplementary Table S1.

## Results

### Characteristics of included studies and patients

A total of 7 studies, published between 2015 and 2023, reporting on 23 individuals with fulminant IIH who underwent venous sinus stenting were included. The studies, which are summarized in Table 1, comprised four case series (including one pediatric series), one study from a prospective registry (with retrospective analysis of fulminant cases), and two single-patient case reports. The number of fulminant IIH patients per study ranged from 1 (case reports) to 10 (the largest series) (20). In aggregate, there were 21 female and 2 male patients (~91% female), consistent with the known female predominance in IIH. The adult patients' mean age was approximately 26 years, while one series focusing on children reported a mean age of 13.4 years (range 4–18) for their pediatric IIH cohort (21). Notably, fulminant IIH can occasionally affect atypical demographics–one included case was a 65-year-old man with “variant IIH” who had an acute, severe presentation (22). Overall, aside from a few older or male outliers, the fulminant cases were largely young individuals, many of whom were obese (when reported) and otherwise similar to typical IIH populations.

**Table 1:** Summary of studies reporting venous sinus stenting in fulminant idiopathic intracranial hypertension.

References	Study design/*N*	Age (yrs), Female (%)	Definition of fulminant IIH	Stent location/Type	Pre-stent gradient (mmHg)	Post-stent gradient (mmHg)	Visualoutcome	Papilledemaoutcome	Headacheoutcome	Complications	Reintervention	Follow-up (mo)
Elder et al. (23)	Retrospective case series (*n* = 4)	27.5 ± 6.3; 100% F	Acute visual loss <4 wk	Transverse–sigmoid; multiple Wallstents	21 ± 4	3 ± 1	3/4 improved, 1 stable	3/4 resolved	4/4 stable/improved	None	None	13 ± 5
Zehri et al. (20)	Retrospective series (*n* = 10)	31.0 ± 9.1; 90% F	Vision decline <8 wk	Transverse–sigmoid; Precise Pro/Wallstent	28.7 ± 6.4	2.2 ± 1.1	8/10 improved	10/10 resolved	5/9 improved	1 (in-stent thrombosis)	2 (restent)	14 (4–36)
Barrero Ruiz et al., (26) (Child's Nerv Syst)	Pediatric case report (*n* = 1)	6; 0% F	Life-threatening ICP crisis	Superior sagittal sinus; Cordis Precise	23	7	Partial improvement	NR	NR	None	1 (LPS)	9
Mugge et al. (24)	Single case (*n* = 1)	22; 100% F	Rapid visual decline over weeks	Right transverse; Wallstent	12	0	Improved to 20/25	Resolved	NR	None	None	6
Monteiro et al. (22)	Case series (*n* = 2)	29 ± 4; 100% F	Acute visual loss	Bilateral transverse; Carotid Wallstent	20 ± 2	3 ± 1	Both improved	Both improved	NR	None	None	12
Krouma et al. (21)	Pediatric series (*n* = 3)	12 ± 2; 66% F	Fulminant pediatric IIH	Transverse–sigmoid; low-profile stents	18 ± 5	4 ± 2	2/3 improved	3/3 resolved	NR	None	1 (restent)	15
Regev et al. (19)	Prospective case series (*n* = 2)	34 ± 5; 100% F	Vision-threatening IIH	Transverse–sigmoid; Venovo stent	25 ± 3	5 ± 2	2/2 improved	2/2 resolved	2/2 improved	None	None	8
Totals/Mean	23 patients	26.6 ± 8.7; 87% F	—	—	—	—	20/23 (87%) improved	21/22 (95%) resolved	11/15 (73%) improved	1/23 (4%)	4/23 (17%)	—

F, female; IIH, idiopathic intracranial hypertension; LPS, lumboperitoneal shunt; NR, not reported; ICP, intracranial pressure. Percentages are calculated from studies reporting extractable data. Follow-up durations are reported as mean or range when available.

### Fulminant IIH definition

The criteria for “fulminant” varied slightly across studies. Two papers explicitly cited the classic definition of fulminant IIH as vision loss progressing within ≤ 4 weeks of symptom onset (20, 23). Others used phrases like “severe acute vision loss” or “rapidly progressive symptoms” over a short span (up to 6–8 weeks) (24). All included patients, by selection, had evidence of an acute threat to vision prompting urgent treatment. For example, Elder et al. (23) selected patients with “severe acute vision loss” at presentation who were all taken for emergent stenting (with temporary CSF diversion). Zehri et al. (20) included IIH patients with “rapidly progressive symptoms…with vision loss,” not all within 4 weeks but all with significant acute visual dysfunction; some had symptom duration up to 6–8 weeks but still worsening quickly. Importantly, all fulminant cases required urgent hospitalization and intervention, distinguishing them from chronic IIH. In summary, although exact time cut-offs differed, each study's included patients manifested markedly accelerated IIH with days-to-weeks of worsening papilledema and vision, warranting the “fulminant” label.

### Presenting features

By definition, severe papilledema and acute vision loss were universal in this cohort. Many patients had Frisen grade IV or V papilledema at presentation and substantial visual field constriction or reduced visual acuity. For instance, in the series by Zehri et al. (20), all 10 patients had either central visual field loss (within central 5°) or significantly decreased Snellen acuity (≤ 20/50 in at least one eye) on presentation. Elder et al. (23) reported initial visual acuities ranging from 20/40 down to counting fingers in their fulminant cases. One case (in Elder et al.'s (23) series) already had optic disc pallor at presentation, indicating chronic damage on top of acute IIH–that patient unfortunately progressed to blindness despite treatment. Patients often had other signs of raised ICP, such as intense headaches (often severe and diffuse) and sometimes pulsatile tinnitus. Headache was noted in almost all cases; in Elder's four patients, all presented with headache along with visual loss. Tinnitus, if present pre-stent, was typically pulsatile and concurrent with the venous sinus stenosis; a few studies specifically noted tinnitus and tracked its improvement (e.g., Zehri et al. (20)). Importantly, neuroimaging in these idiopathic cases was normal except for signs of raised ICP (e.g., flattening of globes and empty sella) and the venous sinus stenosis itself. One unique imaging finding of reversed flow in the superior ophthalmic vein due to venous hypertension was seen in the report by Mugge et al. (24).

### Primary outcomes: ophthalmologic and clinical improvement

**Figure 1:**
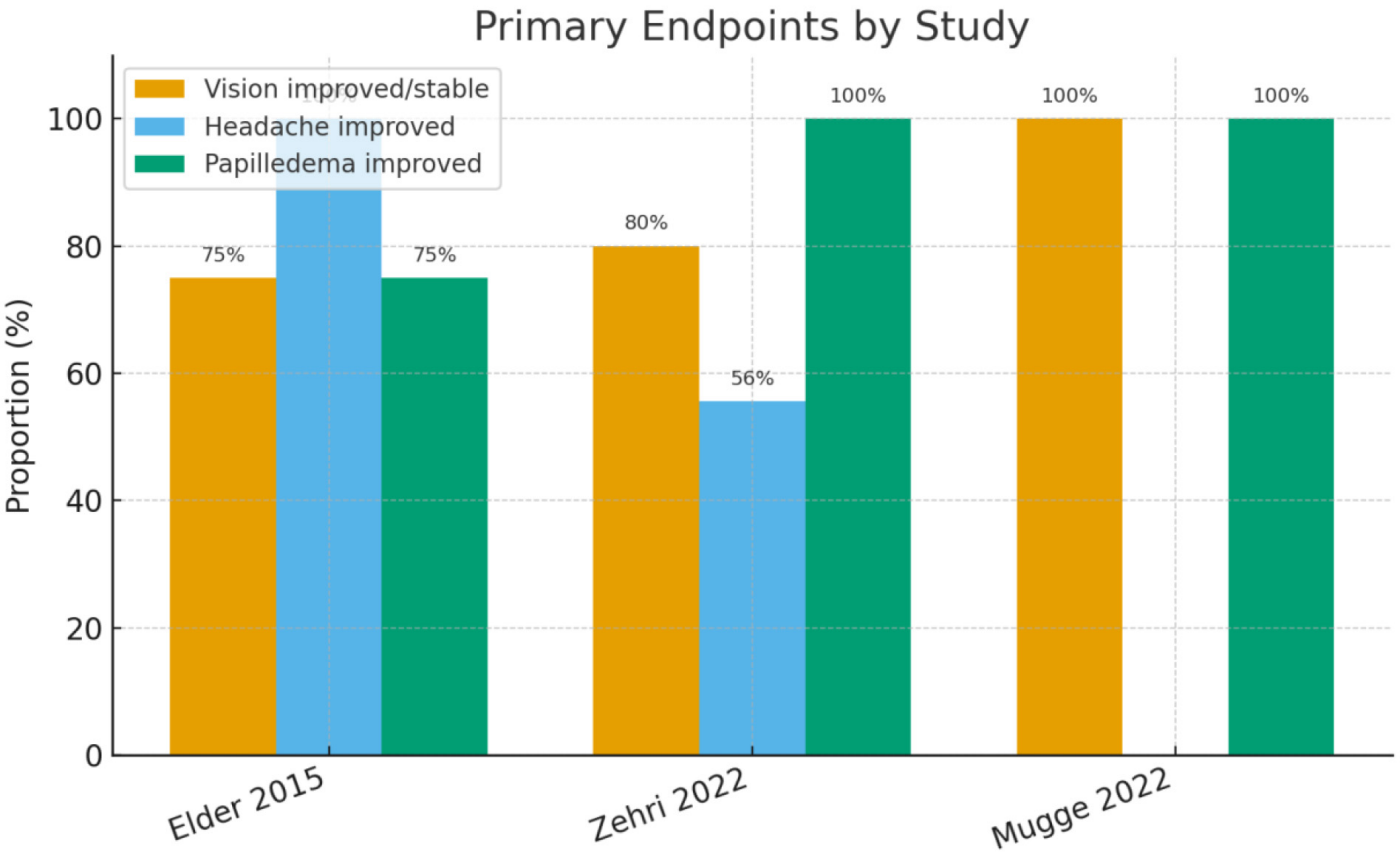
Primary clinical endpoints following venous sinus stenting for fulminant idiopathic intracranial hypertension. Proportion of patients demonstrating improvement or stabilization in **vision**, **headache**, and **papilledema** across the three studies reporting quantitative outcomes (Elder et al. (23), Zehri et al. (20), Mugge et al. (24)). Visual and papilledema improvement were observed in ≥ 75% of patients in all series, while headache improvement ranged from 56–100%. Studies not reporting extractable counts for these endpoints were excluded from this plot.

The main endpoints of interest were improvement in papilledema, visual function, and headaches after VSS, and these are summarized in Figure 1. Across the included studies, nearly all fulminant IIH patients experienced substantial improvement in these outcomes after stent placement (except in cases with irreversible optic nerve damage at the time of treatment).

#### Papilledema

Resolution or significant reduction of optic disc edema was observed in almost every case. Zehri et al. (20) reported that 100% of their 10 patients had improvement in papilledema at mean 14-month follow-up. In that series, Frisen papilledema grades dropped from as high as IV–V at baseline down to 0–1 in most patients by final follow-up. Only one fulminant patient with pre-existing optic atrophy did not show papilledema improvement – because her discs were already atrophic and not edematous (this was the Elder et al. (23) patient who had optic pallor and subsequently went blind).

#### Visual acuity and visual fields

In nearly all 23 patients included in the review, urgent VSS succeeded in preventing further catastrophic vision loss in all cases except for one, who had already presented late in the disease course as mentioned above. Most patients experienced improvement in visual acuity and/or visual field after the stent. Zehri et al. (20) found that 80% of their patients self-reported subjective vision improvement, and objective visual acuity was stable or better in 90% of eyes after stenting. The average post-stent Snellen acuity in their cohort was 20/30, with a mean gain of 3 Snellen lines of vision. They noted some patients improving from 20/200 pre-op to 20/40 or better post-op. Visual field improvements were also recorded: in Zehri et al.'s (20) series, all eyes with initial field loss within the central five degree showed expansion of visual fields after stenting, with visual field mean deviation (MD) improvements an average of 8.57 ± 8.48 (SD) in the worse eye. Elder et al. (23) reported that three of their four patients had improvement in “some or all visual parameters” following stent, implying better visual acuity and/or fields. The one patient who progressed to blindness had presented with hand motions vision and optic disc pallor–presumably beyond salvage by the time of stenting.

#### Headaches and other symptoms

All studies that commented on headaches noted improvement post-stent. Elder et al. (23) reported that headaches were either stable or improved in all four patients by last follow-up (none had worsening or new persistent headaches). Zehri et al. (20) quantified that 55.6% of their patients had significant headache improvement by ~14 months. Pulsatile tinnitus also tended to improve or resolve after stenting. In Zehri et al.'s (20) cohort, 87.5% of those with tinnitus had improvement.

#### Durability and need for further interventions

During available follow-up (which ranged from a few months to a few years, typically 6–24 months), a subset of patients required additional interventions. In Zehri et al.'s (20) series, two out of 10 patients (20%) eventually underwent a second intervention: one had a repeat stent placed due to re-stenosis and another required a CSF shunt for recurrent symptoms, both occurring after the acute phase and after cessation of dual antiplatelets. Other studies did not report significant relapse during their follow-up. Notably, no adult fulminant patient in the included literature ultimately required a shunt or ONSF after having undergone VSS–in all reported adult cases, VSS was sufficient to control the condition without resorting to traditional surgical diversion later.

#### Complications and safety

Venous sinus stenting was generally well-tolerated in these critically ill IIH patients. There was no intracerebral hemorrhage, no vessel perforation, and no permanent neurological deficits related to the procedure reported in any of the studies. The main complication noted was acute in-stent thrombosis in one patient (in the Zehri et al. (20) series). This occurred despite dual antiplatelet loading; the patient developed a thrombus extending from the stent into the superior sagittal sinus during the procedure. The interventionists promptly administered thrombolytic therapy (tPA) through a second catheter and placed another stent to secure access, successfully dissolving the clot. The patient had no stroke or lasting effects and remained thrombosis-free on follow-up. No other stent thromboses were reported in the fulminant cases, which may be related to the strict adherence to DAPT protocols. In summary, the safety profile of VSS in this urgent context appears acceptable and similar to elective cases, with the primary hazard being thrombosis.

### Intervention details (venous sinus stenting procedure)

**Figure 2:**
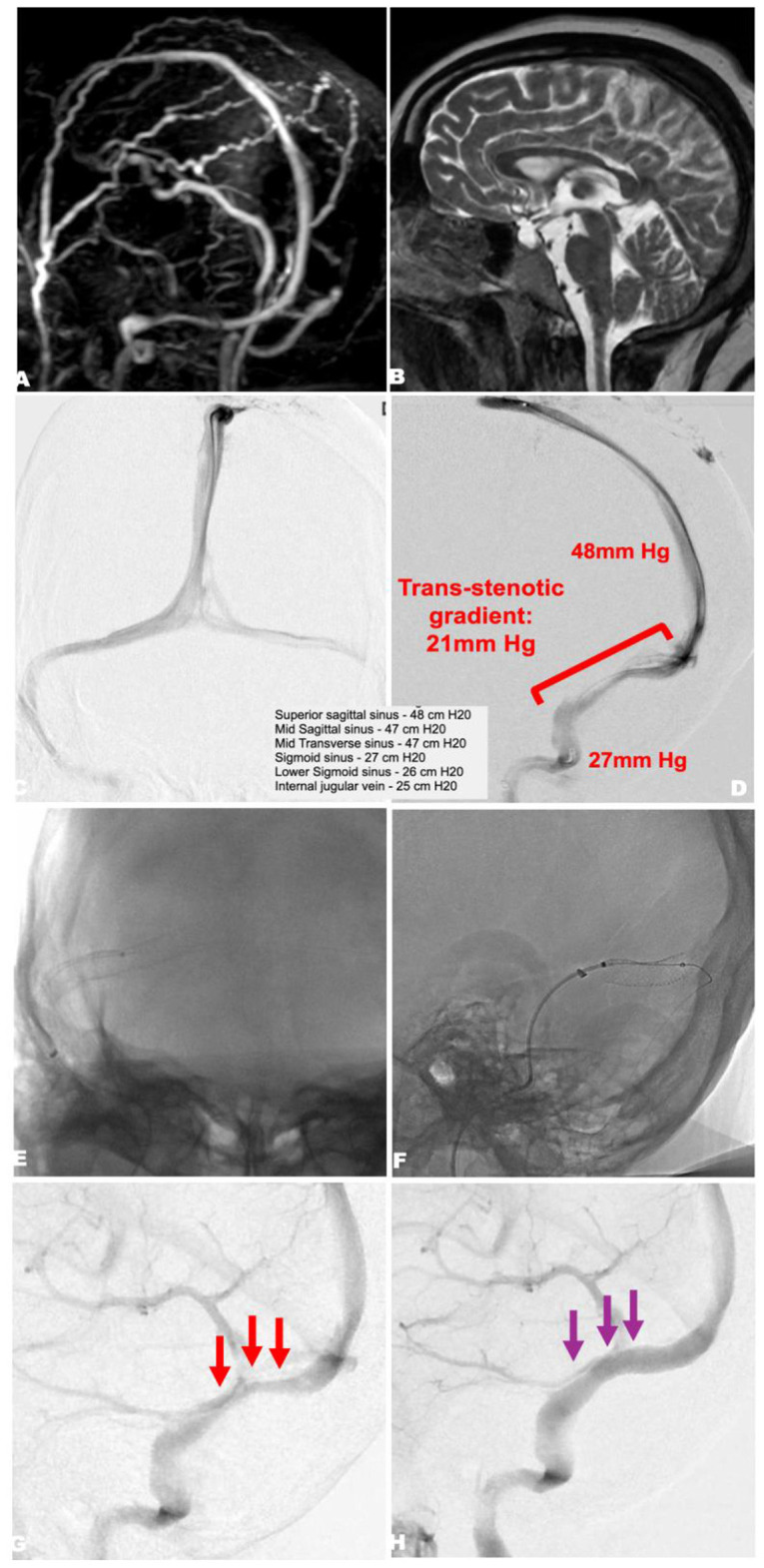
Urgent venous sinus stenting in fulminant idiopathic intracranial hypertension. A 36-year-old obese woman presented with 2 months of progressive vision loss, acutely worsening over 2 weeks. Ophthalmologic examination revealed severe bilateral papilledema (Frisen grade 4 OS, 4+ OD) with visual acuity 20/200 OS and light perception/finger counting OD. Lumbar puncture opening pressure was 46 cm H_2_O. She underwent single-session cerebral angiography, venography, manometry, and venous sinus stenting. **(A)** Time-of-flight MRA demonstrates focal stenosis of the right transverse sinus. **(B)** Sagittal T2-weighted MRI shows brain sag compatible with IIH. **(C, D)** Pre-stent venography and manometry confirm a right transverse–sigmoid sinus stenosis with a trans-stenotic pressure gradient of 21 mm Hg (48 → 27 mm Hg). **(E, F)** Deployment of a self-expanding venous stent across the stenosis from the transverse to sigmoid sinus. **(G)** Pre-stenting angiogram demonstrates flow restriction (red arrows). **(H)** Post-stenting angiogram shows restoration of normal sinus caliber and flow (purple arrows) with elimination of the pressure gradient.

All patients underwent endovascular stenting of a stenosed dural venous sinus, most commonly at the transverse-sigmoid sinus junction on the dominant side. Typically, a single self-expanding stent (8–10 mm diameter range) was deployed across the transverse sinus stenosis into the sigmoid sinus. In fulminant cases, procedures were performed urgently or emergently, often within 48 h of presentation, once the diagnosis and candidacy were confirmed. A representative case treated by the authors in this manner is summarized in Figure 2. Elder et al. (23) describe performing stenting “urgently” with all four patients having a lumbar drain or serial lumbar punctures as a bridge to the procedure. In Zehri et al.'s (20) series, once a patient was identified as fulminant and had venous sinus stenosis on imaging, they performed a venogram and manometry; if a significant pressure gradient was confirmed, patients were loaded with dual antiplatelet medications and stented either the same day or the next morning.

The technical success rate for stenting was 100%–all studies reported successful stent deployment with immediate hemodynamic improvement. Trans-stenotic pressure gradients (measured by catheter manometry) decreased dramatically after stenting in every case in which they were reported. For example, Zehri et al. (20) reported an average pressure gradient of 28.7 mmHg across the stenosis before stenting, which was reduced to essentially 0 mmHg after stent placement. Mugge et al. (24) documented a pre-stent gradient of 12 mmHg in their fulminant case, which normalized to 0 mmHg post-stent, along with resolution of reversed venous flow on angiography. Lumbar puncture opening pressures, when measured after stenting, also showed marked improvement–in Mugge's case, opening pressure went from 70 cm H_2_O before treatment to 21.6 cm H_2_O post-stent.

In general, one stent sufficed for the immediate treatment in all other cases. Antiplatelet therapy was used in all adult patients to prevent in-stent thrombosis. Protocols varied slightly, but most centers loaded patients with aspirin and clopidogrel immediately before stenting (often after the diagnostic venogram) (20). Dual antiplatelet therapy was continued for a period (commonly 3–6 months) after which a single antiplatelet (usually aspirin) was maintained long-term (20).

#### Follow-up duration

Follow-up periods in the studies ranged from a few months up to about 3.5 years. Adult series had 6–18 months follow-up on average.

## Discussion

This systematic review of 23 aggregated patients with fulminant IIH provides preliminary evidence suggesting that VSS may be a safe and effective treatment with respect to core outcome measures of vision preservation, papilledema resolution, and headache improvement. While the traditional paradigm emphasizes urgent surgical intervention (shunt or optic nerve sheath fenestration) is required to prevent irreversible optic neuropathy in fulminant IIH, VSS appears to be a reasonable alternative strategy as nearly all fulminant IIH patients who underwent VSS in this review experienced stabilization or improvement of vision (4, 25). Stenting may be considered early as possible in fulminant IIH, ideally at the first sign of fulminant behavior (e.g., severe papilledema with rapid field loss) and before optic nerve damage becomes irreversible. An expedited work-up for venous sinus stenosis and candidacy for stenting should occur alongside temporizing CSF diversion measures as necessary.

Each treatment modality in the management of fulminant IIH carries inherent risks. For VSS, the main concerns relate to dual antiplatelet therapy and the procedural risks of endovascular intervention as the need for several months of dual antiplatelet therapy can complicate subsequent surgeries, if required. Early stent failure was rare in the reviewed cases—none required shunt placement while on dual therapy. Only one case of acute in-stent thrombosis was reported and was successfully managed endovascularly without sequelae. Across broader IIH series, the rate of in-stent thrombosis is estimated at approximately 1–2% when standard antiplatelet protocols are followed. Major complications such as venous sinus perforation or hemorrhage are also uncommon, and none occurred in the presented fulminant cohort. These findings suggest an acceptable safety profile when procedures are performed by experienced operators. In contrast, CSF shunt surgery carries short-term risks of hemorrhage, infection, or nerve injury (1–5%) and a well-recognized long-term failure rate requiring revisions. ONSF avoids systemic medication use but may fail to arrest vision loss in some cases and carries small risks of orbital hematoma or optic nerve injury. Within these limitations, VSS appears to offer a favorable balance of efficacy and safety, though direct comparative data remain lacking.

Our review carries several limitations. Firstly, all included studies were small, uncontrolled series or case reports, reflecting the rarity of fulminant IIH and the emergent nature of its treatment. Most reports included fewer than five fulminant patients, and the largest series had ten. Selection and publication bias likely favor successful outcomes, and heterogeneous definitions of “fulminant” IIH complicate comparisons. Given the exclusive reliance on uncontrolled case series and case reports, the overall level of evidence remains low (Oxford Centre for Evidence-Based Medicine Level 4), and the findings should be interpreted as hypothesis-generating rather than confirmatory. Follow-up durations were generally short (<2 years), limiting assessment of long-term durability or recurrence and it remains possible that some patients could relapse or require interventions beyond the reported follow-ups. Reporting standards also varied, with some providing detailed ophthalmologic measures while others reported qualitative outcomes. Furthermore, outcome reporting was inconsistent across studies: some provided detailed quantitative ophthalmologic data (e.g., Snellen acuity, visual field mean deviation, and Frisen grades) while others reported only qualitative descriptions of visual improvement. Follow-up durations ranged widely from a few months to over 3 years, further limiting the comparability of outcomes across studies. Notably, the lack of a universally accepted operational definition of fulminant IIH represents a significant limitation both of the existing literature and of this review. We adopted a pragmatic, inclusive definition to capture all relevant cases, but this necessarily introduces heterogeneity in the patient population. Future studies would benefit from consensus-based diagnostic criteria for fulminant IIH to enable more standardized research and meaningful cross-study comparisons.

While direct comparisons to shunting or ONSF are lacking, the consistency of rapid improvement in vision and papilledema across series suggests that VSS is an effective option for appropriately selected fulminant IIH patients. Larger, multicenter registries or prospective studies could help clarify long-term results, restenosis rates, and the optimal timing of stenting relative to medical therapy and permanent CSF diversion. Not withstanding the limitations, VSS appears to be an effective tool to save vision in these urgent cases, but no matter which surgical intervention is utilized, the long term resolution and prevention of recurrences requires significant weight reduction. Clearly, the effectiveness of each therapy is dependent on a multi-disciplinary team with clear communication and decision-making.

## Conclusion

Fulminant idiopathic intracranial hypertension represents a vision-threatening emergency in which rapid intervention is essential. Evidence from published case reports and small series between 2015 and 2025 provides preliminary, hypothesis-generating evidence suggesting that venous sinus stenting (VSS) may offer a promising therapeutic option for selected patients with documented venous sinus stenosis and elevated trans-stenotic pressure gradients. Across studies, VSS was consistently associated with rapid improvement in papilledema, stabilization or recovery of vision, and relief of intracranial pressure–related symptoms, with a low rate of major complications.

Although these exploratory findings suggest that VSS may serve as a sight-saving intervention in fulminant IIH, the current evidence base remains limited by small sample sizes, short follow-up, and the absence of control groups. Further prospective, multicenter data are needed to define long-term outcomes, restenosis rates, and optimal patient selection. Nonetheless, the available literature supports VSS as a feasible and effective option when fulminant IIH presents with venous outflow obstruction, expanding the treatment paradigm beyond traditional surgical approaches such as shunting or optic nerve fenestration.

## Data Availability

The original contributions presented in the study are included in the article/Supplementary material, further inquiries can be directed to the corresponding author.
