# Supplementary material for: Time is vision: a systematic review of urgent venous sinus stenting for fulminant idiopathic intracranial hypertension
Source: Front Neurol. 2026 Apr 30;17:1824887. doi: 10.3389/fneur.2026.1824887 (PMC13174952; doi:10.3389/fneur.2026.1824887)
Supplement: Supplementary file 1 [file Table_1.docx]

**Supplementary Table 1: JBI Critical Appraisal of Included Studies**

*Venous Sinus Stenting for Fulminant Idiopathic Intracranial Hypertension*

## Table 1: JBI Critical Appraisal Checklist for Case Series (3 studies)

| **Study** | **Q1** | **Q2** | **Q3** | **Q4** | **Q5** | **Q6** | **Q7** | **Q8** | **Q9** | **Q10** |
| --- | --- | --- | --- | --- | --- | --- | --- | --- | --- | --- |
| Elder et al. 2015 | Yes | Yes | Yes | Unclear | Unclear | Yes | Yes | Yes | No | N/A |
| Zehri et al. 2022 | Yes | Yes | Yes | Yes | Yes | Yes | Yes | Yes | No | Yes |
| Regev et al. 2025 | Yes | Yes | Yes | Unclear | Unclear | Partially | Yes | Yes | No | N/A |
| **Overall Quality** | **3/3** | **3/3** | **3/3** | **2/3** | **2/3** | **2/3** | **3/3** | **3/3** | **0/3** | **1/3** |

*Legend: Q1 = Clear inclusion criteria; Q2 = Standardized condition measurement; Q3 = Valid identification methods; Q4 = Consecutive inclusion; Q5 = Complete inclusion; Q6 = Clear demographic reporting; Q7 = Clear clinical information; Q8 = Clear outcome reporting; Q9 = Site demographic reporting; Q10 = Appropriate statistical analysis.*

## Table 2: JBI Critical Appraisal Checklist for Case Reports (4 studies)

| **Study** | **Q1** | **Q2** | **Q3** | **Q4** | **Q5** | **Q6** | **Q7** | **Q8** |
| --- | --- | --- | --- | --- | --- | --- | --- | --- |
| Mugge et al. 2022 | Yes | Yes | Yes | Yes | Yes | Partially | Yes | Yes |
| Krouma et al. 2025 | Yes | Yes | Yes | Yes | Yes | Yes | Yes | Yes |
| Monteiro et al. 2023 | Partially | Yes | Yes | Yes | Yes | Yes | Yes | Yes |
| Barrero Ruiz et al. 2022 | Yes | Yes | Yes | Yes | Yes | Partially | Yes | Yes |
| **Overall Quality** | **3/4** | **4/4** | **4/4** | **4/4** | **4/4** | **3/4** | **4/4** | **4/4** |

*Legend: Q1 = Clear demographic description; Q2 = Clear clinical timeline; Q3 = Clear presentation description; Q4 = Clear diagnostic testing; Q5 = Clear intervention description; Q6 = Clear post-intervention condition; Q7 = Adverse events identified; Q8 = Takeaway lessons provided.*

**Methods**

Critical appraisal was conducted using the Joanna Briggs Institute (JBI) standardized checklists for case series and case reports. The JBI checklist for case series comprises 10 items assessing study design clarity, inclusion criteria, measurement methods, and outcome reporting. The JBI checklist for case reports comprises 8 items evaluating demographic and clinical presentation clarity, diagnostic testing, intervention details, follow-up outcomes, adverse events, and clinical relevance. Each item was rated as "Yes" (criterion met), "No" (criterion not met), "Unclear" (insufficient information), or "Partially" (criterion partially met). Studies meeting more criteria received higher quality ratings.
